# Local Pressure of Supercritical Adsorbed Hydrogen in Nanopores

**DOI:** 10.3390/ma11112235

**Published:** 2018-11-10

**Authors:** Jimmy Romanos, Sara Abou Dargham, Roy Roukos, Peter Pfeifer

**Affiliations:** 1 Department of Natural Sciences, Lebanese American University, P.O. Box 36, Byblos, Lebanon; sara.aboudargham@lau.edu.lb (S.A.D.); roy.roukos@gmail.com (R.R.); 2 Department of Physics, University of Missouri, Columbia, MO 65201, USA; pfeiferp@missouri.edu

**Keywords:** hydrogen storage, ono-kondo, activated carbon, adsorption, nanopores

## Abstract

An overview is given of the development of sorbent materials for hydrogen storage. Understanding the surface properties of the adsorbed film is crucial to optimize hydrogen storage capacities. In this work, the lattice gas model (Ono-Kondo) is used to determine the properties of the adsorbed hydrogen film from a single supercritical hydrogen isotherm at 77 K. In addition, this method does not require a conversion between gravimetric excess adsorption and absolute adsorption. The overall average binding energy of hydrogen is 4.4 kJ/mol and the binding energy at low coverage is 9.2 kJ/mol. The hydrogen film density at saturation is 0.10 g/mL corresponding to a local pressure of 1500 bar in the adsorbed phase.

## 1. Introduction

The standard physical-based methods for the transport and storage of hydrogen are compressed, liquefied, and cryo-compressed hydrogen. Hydrogen storage in materials emerged as a safe and practical alternative for vehicles. Hydrogen can be stored by physisorption or chemisorption in materials. In chemisorption, hydrogen is absorbed in metal hydrides through ionic, covalent, or metallic-type bonds. Chemical hydrides are liquids or solids which release hydrogen when heated or exposed to a catalyst [1,2]. However, chemisorption is not reversible. Metal hydrides release hydrogen at high temperature and chemical hydrides require recycling for each charging cycle. In physisorption, hydrogen molecules are attracted to sorbents through weak van der Waals forces, without developing a chemical bond. Activated carbon (AC), zeolites, and metal-organic framework (MOF) are the leading materials for reversible hydrogen storage [3,4,5,6,7,8,9]. However, hydrogen storage capacities at ambient temperatures are relatively low due to the weak binding energy. In fact, the heat of hydrogen adsorption in such materials is in the range of about 4–8 kJ/mol [10,11,12,13,14,15].

**Figure 1:**
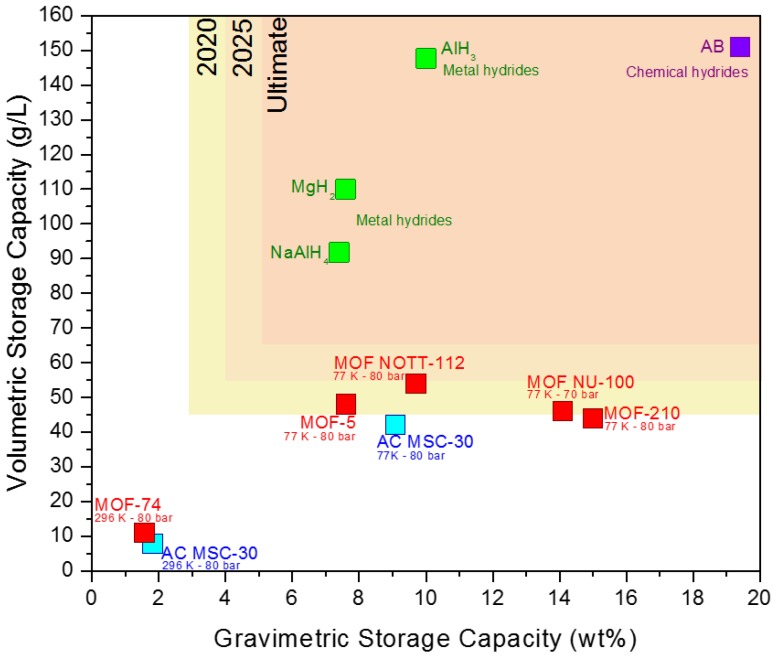
Volumetric vs. gravimetric storage capacities in MOFs (red) and activated carbon (blue). Metal (green) and chemical (violet) hydrides data are calculated based on the atomic composition.

The US Department of Energy (DOE) has set the 2020, 2025, and ultimate hydrogen storage targets listed in Figure 1 [16]. Despite tremendous research efforts, no material developed until now meets the technical system target set by US DOE. It is important to differentiate between material and system storage capacities. While many materials in Figure 1 meet the ultimate DOE target, the system-based storage is still below the DOE target. For instance, when the mass and volume of the engineering components (tank, pipes, valves, vents, etc.) are considered, all storage metrics will fail to meet the DOE targets.

Increasing the hydrogen storage capacities of an adsorbent material to meet the DOE targets requires a high specific surface area and an optimal binding energy of 15 kJ/mol [17]. A low binding energy results in low storage capacities and a high binding energy results in non-reversible storage as in the case of chemisorption. In our previous work, we have discussed methods to optimize the binding energy by modifying the surface chemistry and the pore size distribution [18,19]. The binding energy of hydrogen is usually determined using two isotherms at nearby temperatures using Clausius-Clapeyron equation. The determination of the experimental binding energy of hydrogen has been a challenge since it requires a conversion from experimental excess adsorption to absolute adsorption using the volume of the adsorbed film [20]. Firlej et al. proposed a method to determine the high-coverage binding energy using a single isotherm and the pore-size distribution [21]. Aranovich and Donohue were the first to explore the Ono-Kondo model for slit-shaped pores [22]. Chahine et al. applied it to supercritical gas using a linear fit to excess adsorption [23]. In this work, the average binding energy at low coverage and the overall average binding energy are extracted from a single supercritical hydrogen adsorption isotherm using the lattice gas model and chi-squarre minimization algorithm. In addition, the film density at maximum capacity and the local pressure in nanopores are determined using the Ono-Kondo model.

## 2. Material and Methods

**Figure 2:**
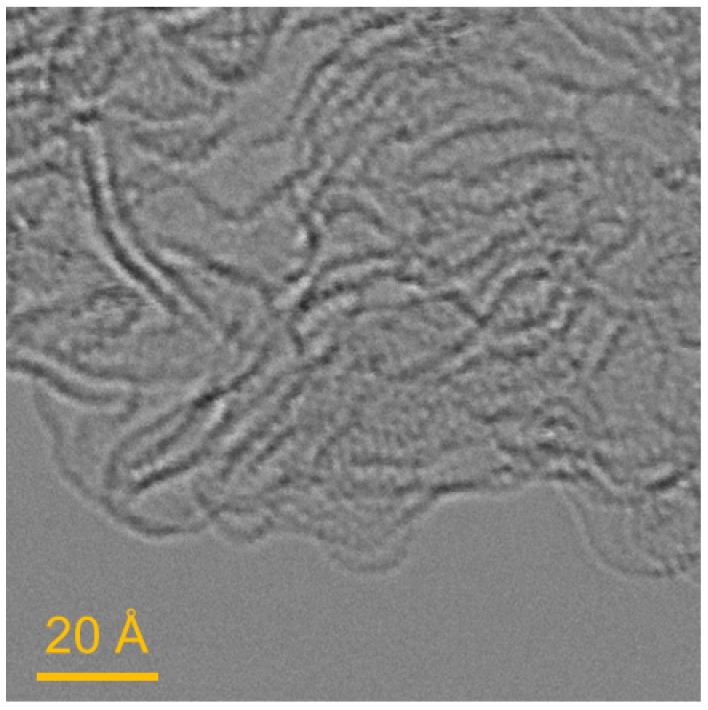
STEM micrograph of MSC-30.

A commercial high-surface area carbon Maxsorb MSC-30 [24], produced by Kansai Coke and Chemical Co., Ltd. (Hyogo, Japan) have been selected for this study. Samples are prepared for scanning transmission electron microscopy (STEM) by dispersing the carbon in methanol and depositing it on holey carbon support film. STEM micrographs in Figure 2 are taken using were performed on a Nion UltraSTEM 100 with a third-generation C3/C5 aberration correction. 60 keV was used to avoid beam damage on carbon. Figure 2 shows that activated carbon grains are formed of corrugated carbon sheets with typical dimensions of 2–5 nm. The absence of graphitic components indicates that the carbon has been completely activated. Consequently, using the slit-shaped pores structure for MSC-30 is a suitable model for hydrogen adsorption on activated carbon.

Nitrogen adsorption isotherms were measured on an Autosorb instrument (Quantachrome, Boynton Beach, FL, USA). The total pore volume (V*_tot_*) is measured at a pressure of 0.995 P/P_0_ while the specific surface area Σ is measured using the Brunauer-Emmett-Teller (BET) theory in the pressure range of 0.01 to 0.03 P/P_0_, which is suitable for nanoporous activated carbon. The surface area was rounded to the nearest hundred. The intragranular porosity is calculated as follows:

(1) $$\begin{array}{c} \begin{array}{c} \phi ={\left[1+{\left(\rho_{skel}\cdot \frac{V_{tot}}{m_{s}}\right)}^{-1}\right]}^{-1} \end{array} \end{array}$$

where $\rho_{skel}=2$ g/cm^3^ is the skeletal density of activated carbon. The porosity ϕ is calculated from the total pore volume (V*_tot_*) using Equation (1).

(2) $$\begin{array}{c} \begin{array}{c} \rho_{app}=\rho_{skel}\left(1-\phi \right) \end{array} \end{array}$$

The apparent density $\rho_{app}$ is determined using Equation (2). Quenched solid-density functional theory (QSDFT) for infinite slit-shaped pores is used to calculate the pore-size distribution. QSDFT is a modified version of the non-local density functional theory (NLDFT). NLDFT, which assumes a flat graphitic pore structure, has a significant drawback when applied to activated carbons where heterogeneities obstruct layering transitions, thus leading to false minimums in the pore-size distribution. This artifact has been completely eliminated with QSDFT by considering surface roughness and heterogeneity. The surface is modeled using the distribution of solid atoms rather than the source of the external potential field [25,26]. As a consequence, the resulting QSDFT pore-size distributions are more reliable for high-surface area carbon.

**Figure 3:**
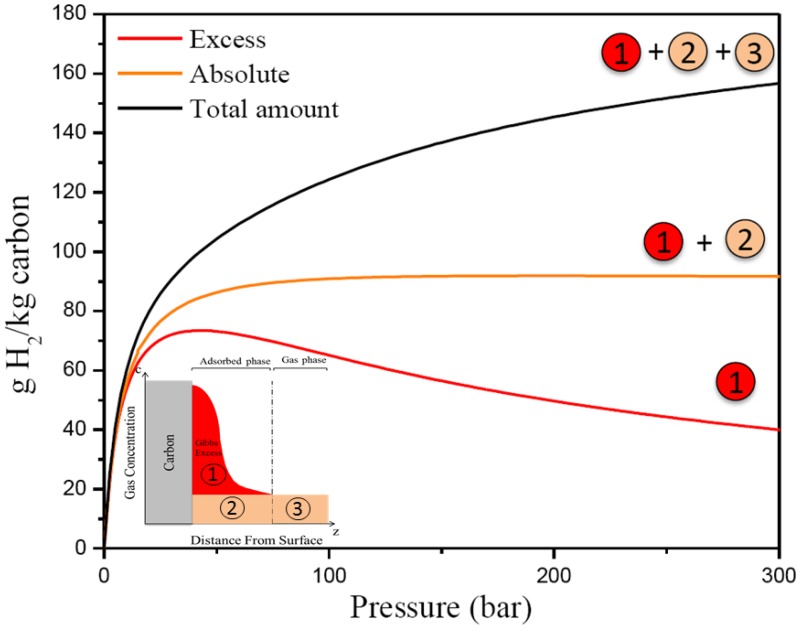
Excess adsorption (red), absolute adsorption (orange), and total gravimetric storage capacity (black).

The performance of an adsorbent is often measured by collecting an excess adsorption isotherm and converting that into gravimetric and volumetric storage capacities by knowing the pore volume of the adsorbent. Gravimetric excess adsorption is the mass of the adsorbed film minus the mass of an equal volume of gas [27,28]. The output of computational adsorption isotherms is the absolute adsorption. For practical engineering applications, the total storage capacity is the metric of interest. Figure 3 shows the excess adsoprtion, absolute adsorption, and total storage capacity as a function of pressure. Methane excess adsorption measurements at 35 bar and ambient temperature were performed using a custom-built Sievert apparatus described extensively in literature [29,30,31].

Aranovich and Donohue extended the Ono-Kondo model to gas adsorption on activated carbon [22]. It was applied to supercritical fluid by Chahine et al. [23]. and more recently by Gasem et al. [32]. The excess adsorption, determined from solving Ono-Kondo equations for slit shaped pores, has four parameters: energy of the adsorbate-adsorbate interaction $E_{H2-H2}$ (K), energy of adsorbate-adsorbent interaction *E* (K), density of the adsorbed film at maximum capacity $\rho_{mc}$ (g/mL), and a prefactor *C* related to the capacity of the adsorbent for a specific gas. Aranovich and Donohue solved Ono-Kondo equations which relate the density of each layer to the bulk density and found the following general equation for the excess adsorption. Moreover, by neglecting the gas-gas interaction, one can reduce the number of parameters to three:

(3) $$\begin{array}{c} \begin{array}{c} G_{e}\left(P,T\right)=2C\frac{1-w^{n}}{\left(1-w_{1}\right)\left(1+w_{1}^{n-1}\right)}\frac{1-\frac{\rho_{gas}\left(P,T\right)}{\rho_{mc}}\left(1-e^{\frac{E}{KT}}\right)}{1+\left(\frac{\rho_{mc}}{\rho_{gas}\left(P,T\right)}-1\right)e^{\frac{E}{KT}}} \end{array} \end{array}$$

where $G_{e}\left(P,T\right)$ is the gravimetric excess adsorption, $\rho_{gas}\left(P,T\right)$ is the density of hydrogen at pressure *P* and temperature *T*, *n* is the number of layer in a slit of the microporous material, $w_{1}$ is a factor which is a function of three coordination number and the fluid-fluid interaction energy and other variables discussed in detail by Aranovich and Donohue. For n=2, the excess adsorption can be written as:

(4) $$\begin{array}{c} \begin{array}{c} G_{e}\left(P,T\right)=2C\frac{1-\frac{\rho_{gas}\left(P,T\right)}{\rho_{mc}}\left(1-e^{\frac{E}{KT}}\right)}{1+\left(\frac{\rho_{mc}}{\rho_{gas}\left(P,T\right)}-1\right)e^{\frac{E}{KT}}} \end{array} \end{array}$$

Chi-square Levenberg-Marquardt minimization algorithm was used to fit experimental excess adsorption with Equation (2) for the Ono-Kondo Model.

## 3. Results and Discussion

**Figure 4:**
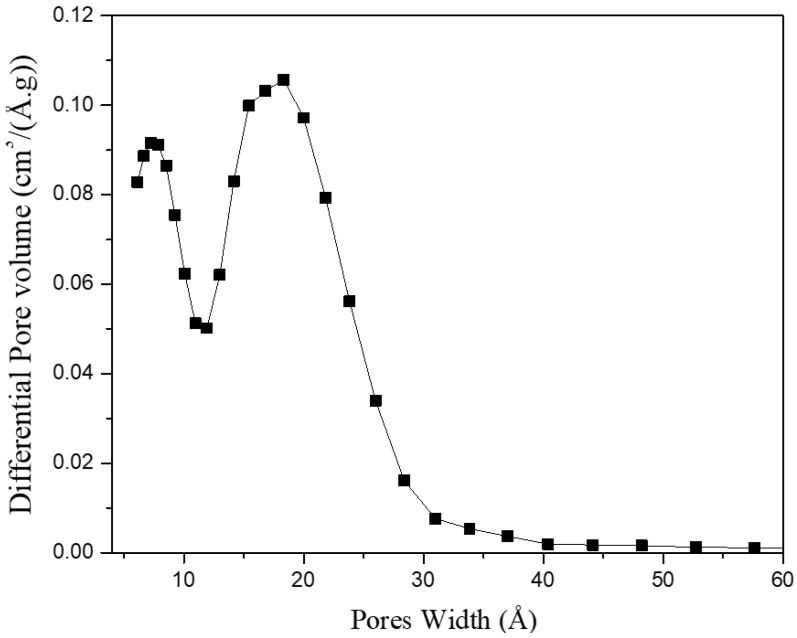
Pore size distribution of MSC-30 from QSDFT.

**Figure 5:**
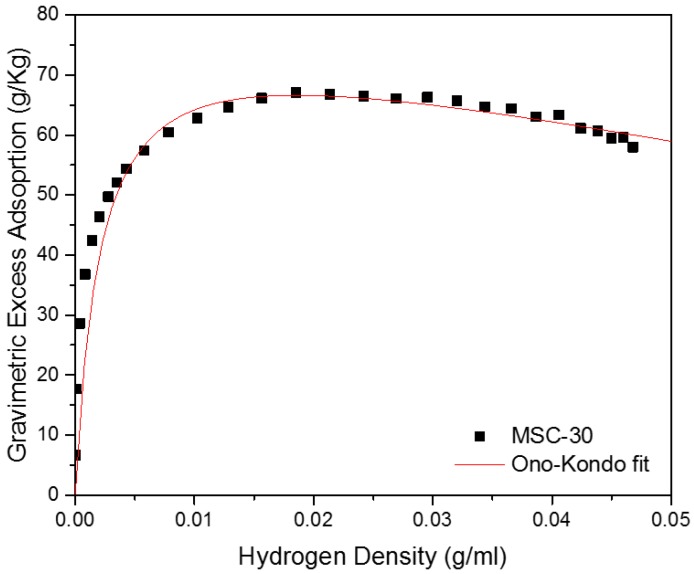
Ono-Kondo fit of the hydrogen Excess Adsorptionfor MSC-30.

**Figure 6:**
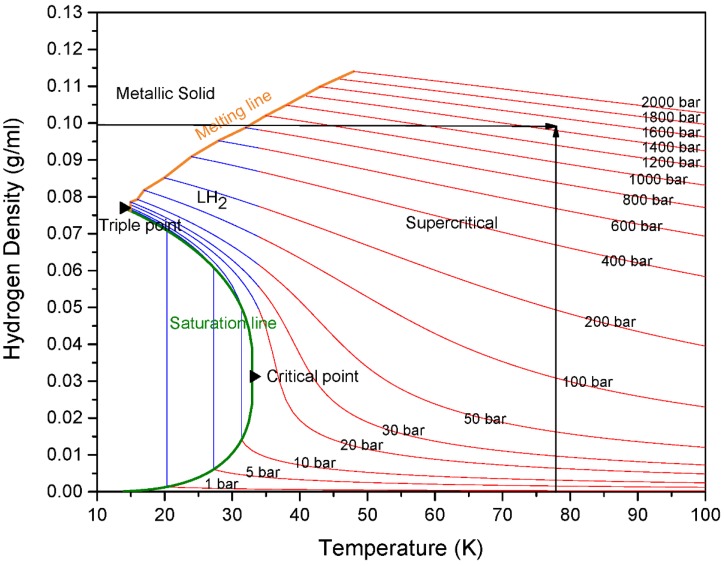
Phase diagram showing the hydrogen density as a function of temperature at different pressures.

MSC-30 has BET surface area Σ=2700 m^2^/g and an intragranular porosity ϕ=0.77. The large surface area indicates the absence of non-activated graphitic components and validates that MSC-30 is mainly formed of graphene sheets oriented in random direction. The width of the pores represents the separation between two graphene sheets. MSC-30 has pores smaller than 40 Å. In addition, Figure 4 shows the sample has a bimodal pore size distribution with peak at 7 Å and another peak at 20 Å. Consequently, using the lattice gas model for slit-shaped pores is a valid approximation for high-surface area activated carbon. Figure 5 shows the Ono-Kondo fit for MSC-30. This model provide the average binding energies from a single isotherm. Most binding-energy determinations need two isotherms at nearby temperatures (Clausius-Clapeyron), and those are known to be tricky at high coverage because it requires a good estimates of the film volume to construct accurate absolute adsorption isotherms. As a consequence, there exist only a few accurate determinations of high-coverage binding energies. The only other single-isotherm method to get an average or high-coverage binding energy requires the knowledge of the pore-size distribution [21]. In contrast, this model provide the average binding energy accurately from one single fit to the excess adsorption isotherm. In addition, it does not require a conversion between gravimetric excess adsorption and absolute adsorption. The overall average binding energy of hydrogen on MSC-30 is 4.43 ± 0.40 kJ/mol. This is comparable to the reported binding energies between 4 and 8 kJ/mol of hydrogen on carbon [21]. In addition, the average binding energy is 9.2 ± 0.74 kJ/mol at low coverage, which is obtained by fitting the excess adsorption at densities below 0.01 g/mL. The results obtained in this work match the ones obtained from existing methods. Using Clausius-Clapeyron equation, Purewal et al. reports an average heat of adsorption of 8–9 kJ/mol at low coverage and 6 kJ/mol at complete coverage for activated carbon [33]. In addition, Schmitz et al. measured an average heat of adsorption between 4 and 5.7 kJ/mol for several sorbent materials [34]. The hydrogen film density at saturation is 0.10 ± 0.06 g/mL, larger than the liquid density of hydrogen at subcritical temperatures. Figure 4 shows the phase diagram of hydrogen as a function of temperature. The hydrogen density in Figure 6 is from the National Institute of Standards and Technology (NIST) Thermophysical Properties of Fluid [35]. At 80 K, the density of the adsorbed film is equivalent to 1500 bar. Hydrogen molecule in the adsorbed phase are subject to local pressures of 1500 bar due to the average overall binding energy of 4.4 kJ/mol. While the pressure in the gas phase is between 0 and 200 bar, hydrogen molecules are subject to a pressure of 1500 bar in the adsorbed phase. This pressure represents the combined average normal and tangential pressure on the carbon surface. Normal and tangential pressure due to van der Waals force between Argon gas and graphene in slit-shaped pores has been approximated between 10^3^ and 10^4^ bar [36]. Phases confined in nanospaces exhibit a behavior that is different behavior from that of the bulk phase. Such difference arise from reduced dimenionality and from the van der Waals interaction of the adsorbate molecules with the walls of the porous materials.

## 4. Conclusions

In summary, we presented a method to determine the properties of the adsorbed hydrogen film from a single supercritical isotherm. This method does not require a conversion between gravimetric excess adsorption and absolute adsorption. The average binding energy of hydrogen on nanoporous carbon is 9.2 kJ/mol at low coverage. In addition, the overall average binding energy is 4.4 kJ/mol. The hydrogen film density at saturation is 0.10 g/mL, which corresponds to a local pore pressure of 1500 bar.
